# Safety and surgical outcomes of robotic adrenalectomy from a 15-year experience at a single institution

**DOI:** 10.1038/s41598-024-63105-9

**Published:** 2024-05-28

**Authors:** Kwangsoon Kim, Dawon Park, Moon Young Oh, Young Jun Chai, Hoon Yub Kim

**Affiliations:** 1 Department of Surgery, Korea University College of Medicine, Seoul, Republic of Korea; 2 Department of Surgery, College of Medicine, The Catholic University of Korea, Seoul, Republic of Korea; 3 Department of Surgery, Seoul National University College of Medicine, Seoul Metropolitan Government-Seoul National University Boramae Medical Center, Seoul, Republic of Korea

**Keywords:** Robotic surgery, Adrenalectomy, Robotic adrenalectomy, Surgical outcome, Endocrinology, Adrenal glands

## Abstract

Robotic adrenalectomy (RA) has gained significant popularity in the management of adrenal gland diseases. We report our experience at a single tertiary institution and evaluate the safety and surgical outcomes of RA. The data of 122 consecutive patients who underwent RA from October 2009 to December 2022 at Korea University Anam Hospital (Seoul, Korea) were reviewed. There were no perioperative complications. Clinicopathological features and surgical outcomes were retrospectively analyzed through complete chart reviews. Noteworthy findings include the influence of sex, tumor size, and body mass index on operation time, with the female and small tumor groups exhibiting shorter operation times (*P* = 0.018 and *P* = 0.009, respectively). Pheochromocytoma was identified as a significant independent risk factor for a longer operation time in the multivariate analysis [odds ratio (OR), 3.709; 95% confidence interval (CI), 1.127–12.205; *P* = 0.031]. A temporal analysis revealed a decreasing trend in mean operation times across consecutive groups, reflecting a learning curve associated with RA adoption. RA is a safe and effective operative technique alternative to laparoscopic adrenalectomy that has favorable surgical outcomes and enhances the convenience of the operation.

## Introduction

In recent decades, the field of surgery has experienced a remarkable transformation, driven by advancements in robotic technology and an evolving understanding of endocrine disorders^1,2^. One notable innovation is robotic adrenalectomy (RA), a minimally invasive surgical approach that has gained significant popularity in the management of adrenal gland diseases^1,3,4^. The adrenal gland plays a crucial role in regulating numerous physiological functions by releasing hormones, such as cortisol, aldosterone, and adrenaline. Dysregulation of adrenal gland function can result in various diseases, including Cushing’s syndrome, primary hyperaldosteronism, and pheochromocytoma, among others^5^.

Adrenalectomy is the gold standard for most adrenal gland diseases^6,7^. However, laparoscopic adrenalectomy, first performed by Gagner in 1992, gained widespread adoption in subsequent years and due to its favorable outcomes, is now the established standard technique^8–13^. The benefits of laparoscopic adrenalectomy include reduced pain, shorter hospitalization, and faster recovery. However, surgeons have transitioned to RA because of its inherent benefits, including advanced three-dimensional visualization, increased flexibility with possible 360° rotation, improved depth perception, enhanced dexterity, and superior ergonomics^14–16^. Consequently, this technique enables precise resection of the adrenal gland, which requires meticulous dissection along major vessels and organs, as well as delicate dissection in limited spaces, such as the retroperitoneum.

Robotic adrenal surgery is an exciting development that can improve patient outcomes, reduce postoperative complications, and enhance quality of life. Additionally, numerous studies have proven the safety and feasibility of RA^17–20^. Compared with laparoscopic adrenalectomy, RA is associated with lower blood loss, reduced patient morbidity, and shorter hospital stays^3,18,21^.

With an increasing interest in minimally invasive surgery and a growing emphasis on optimizing patient outcomes, RA has also emerged as a promising approach for treating adrenal diseases. In this study, we share our experience with RA and evaluate the safety and surgical outcomes of RA in a consecutive series of 122 cases.

## Methods

### Patients

We conducted a retrospective analysis of 122 consecutive patients who underwent RA at Korea University Anam Hospital from October 2009 to December 2022. All procedures were performed by a single surgeon (H.Y.K.), with extensive experience in RA who had performed more than 200 cases with laparoscopic adrenalectomy. Out of 122 patients, 113 (92.6%) cases underwent lateral transperitoneal adrenalectomy (LTA), and 9 (7.4%) cases underwent posterior retroperitoneoscopic adrenalectomy (PRA). All patients were preoperatively diagnosed with non-functioning lesions, primary hyperaldosteronism, pheochromocytoma, or Cushing’s syndrome, using hormonal tests, and radiological examinations. The average postoperative hospital stay was 5 ± 2 (range, 3–12) days.

We retrospectively collected clinical and pathological data, including age, gender, height, weight, body mass index (BMI), hospital stay, type of disease, operation time, and postoperative pain score using visual analog scale (VAS). Overweight was defined as a BMI of 25 kg/m^2^ or higher according to the World Health Organization (WHO) and the International Association for the Study of Obesity (IASO)^22^. WHO and IASO define obesity as a BMI of 30 or above^23^. However, Asian countries have lower cutoff values due to a higher prevalence of obesity-related diseases at lower BMI levels. As this study included Korean individuals, the patients were divided by a BMI of 25, which is the standard for overweight defined by WHO and for obesity in Asia^24^. The VAS pain scores were recorded at 4, 24, 48, and 72 h after surgery. This study was conducted in accordance with the Declaration of Helsinki (as revised in 2013) and approved by the Institutional Review Board at Korea University Hospital (IRB No.: 2023AN0093), which waived the requirement for informed consent due to the retrospective nature of the study.

### Operative procedures: LTA

Comprehensive surgical procedures and techniques for LTA have been previously described^25–27^. Briefly, the patient is positioned at a 45-degree lesion-side-up position for LTA. An initial incision is made near the umbilicus, through which a needle is inserted into the abdomen. Then, carbon dioxide gas is injected into the abdominal cavity to create a working space. A robotic camera is inserted through the umbilical incision. The surgeon makes additional small incisions in the abdomen and inserts specialized robotic instruments, such as a prograsper, forceps, and an energy device, for precise manipulation of the adrenal gland. Working from the console, the surgeon delicately dissects the adrenal gland from the surrounding tissues, blood vessels, and adrenal vein. Then, the adrenal vein is ligated or clipped to prevent bleeding, and the gland is removed from the body. It is worth noting that there are variations in the surgical procedures and techniques between right and left LTA.

### Operative procedures: PRA

The detailed surgical procedures and techniques for PRA have been described elsewhere^28,29^. Briefly, the patient is placed in a prone jackknife position with their hip joints bent and fixed at a right angle. Soft pillows and pads are applied at the weight bearing and bony prominent areas to avoid the direct pressure. The surgeon makes a small incision in the back and creates a space between the retroperitoneum and the muscles of the back. The surgeon makes additional small incisions in the back or flank region and inserts specialized robotic instruments. Working from the console, the surgeon carefully dissects the adrenal gland from the surrounding tissues, blood vessels, and adrenal vein. The adrenal vein is then ligated or clipped to prevent bleeding, and the gland is removed from the body.

### Statistical analysis

All statistical analyses were conducted using the SPSS software package (version 24.0; IBM Corp., Armonk, NY, USA). Continuous variables were expressed as means with corresponding standard deviations, while categorical variables were presented as counts and percentages. Continuous variables were compared using Student’s t-tests, and categorical characteristics were compared using Pearson’s chi-square tests or Fisher’s exact tests, as appropriate. Multivariate logistic regression analyses were carried out to validate which factors were associated with operation time. Odds ratios (ORs) with 95% confidence intervals (CIs) were calculated to compare the risk of operation time between the independent factors by using linear logistic regression analysis. Statistical significance was defined as p-values < 0.05.

## Results

### Baseline clinicopathological characteristics

Table 1 presents the baseline clinicopathological characteristics of the 122 patients who underwent RA for adrenal gland diseases. The mean age was 50.3 ± 13.7 (range, 19–74) years, and 72 patients (59%) were female. The mean BMI was 24.8 ± 4.2 (range, 17.2–44.3) kg/m^2^, and 52 (42.6%) patients were classified as overweight. The mean tumor size was 2.8 ± 1.5 (range, 0.8–10.1) cm. Most patients (113 patients, 92.6%) underwent LTA, and 9 (7.4%) patients underwent posterior retroperitoneoscopic adrenalectomy (PRA). Thirty-six (29.5%) patients underwent right-side surgery, whereas 86 (70.5%) patients underwent left-side surgery. Adrenal tumor characteristics included non-functioning lesions, primary hyperaldosteronism, pheochromocytoma, and Cushing’s syndrome, with each accounting for 59.8%, 13.1%, 16.4%, and 10.7%, respectively. In most cases, RA for non-functioning lesions was at the patient's request, while for some, it was to diagnose metastasis of accompanying cancers. Among them, 71 patients (97.2%) were diagnosed with myelolipoma, and 2 patients (2.8%) were diagnosed with ganglioneuroma.

**Table 1:** Baseline clinicopathologic characteristics of the study population.

Total 122 patients	
Age (years)	50.3 ± 13.7 (range, 19–74)
Gender	
Male	50 (41.0%)
Female	72 (59.0%)
BMI (kg/m^2^)	24.8 ± 4.2 (range, 17.2–44.4)
Overweight	52 (42.6%)
Tumor size (cm)	2.8 ± 1.5 (range, 0.8–10.1)
Surgical approach	
LTA	113 (92.6%)
PRA	9 (7.4%)
Operation side	
Right	36 (29.5%)
Left	86 (70.5%)
Surgical period	
2009–2018	63 (51.6%)
2019–2022	59 (48.4%)
Preoperative diagnosis	
Non-functioning lesions	73 (59.8%)
Primary hyperaldosteronism	16 (13.1%)
Pheochromocytoma	20 (16.4%)
Cushing’s syndrome	13 (10.7%)

Data are expressed as the patient number (%) or mean ± SD.

*BMI* body mass index, *LTA* lateral transperitoneal adrenalectomy, *PRA* posterior retroperitoneoscopic adrenalectomy.

## Comparison of baseline clinicopathological characteristics according to sex, tumor size, and BMI

Table 2 shows the baseline clinicopathological characteristics according to sex. The female group was significantly younger than the male group (47.7 ± 12.9 vs. 54.0 ± 13.9 years, *P* = 0.011). The mean operation time was significantly shorter for the female group than for the male group (137.4 ± 46.6 vs. 158.6 ± 49.5 min, *P* = 0.018). However, the BMI, mean tumor size, surgical approach, operative side, preoperative diagnosis, and postoperative VAS pain score did not significantly differ between the groups.

**Table 2:** Baseline clinicopathological characteristics according to gender.

	Male (n = 50)	Female (n = 72)	p-value
Age (years)	54.0 ± 13.9	47.7 ± 12.9	0.011
BMI (kg/m^2^)	25.2 ± 3.3	24.5 ± 4.7	0.375
Overweight	24 (48%)	28 (38.9%)	0.355
Operation time (min)	158.6 ± 49.5	137.4 ± 46.6	0.018
Tumor size (cm)	3.0 ± 1.8	2.7 ± 1.2	0.300
Surgical approach			0.485
LTA	45 (90%)	68 (94.4%)	
PRA	5 (10%)	4 (5.6%)	
Operation side			0.921
Right	15 (30%)	21 (29.2%)	
Left	35 (70%)	51 (70.8%)	
Surgical period			0.582
2009–2018	24 (48%)	39 (54.2%)	
2019–2022	26 (52%)	33 (45.8%)	
Preoperative diagnosis			0.577
Non-functioning lesions	28 (56%)	45 (62.5%)	
Primary hyperaldosteronism	8 (16%)	8 (11.1%)	
Pheochromocytoma	10 (20%)	10 (13.9%)	
Cushing’s syndrome	4 (8%)	9 (12.5%)	
Postoperative VAS			
POD 0	5.0 ± 1.6	5.1 ± 1.8	0.743
POD 1	3.6 ± 1.1	3.4 ± 1.1	0.490
POD 2	3.2 ± 1.1	3.0 ± 0.9	0.430
POD 3	2.9 ± 0.9	2.7 ± 0.9	0.072

Data were expressed as number (%) or mean ± standard deviation.

A statistically significant difference was defined as p < 0.05.

*BMI* body mass index, *LTA* lateral transperitoneal adrenalectomy, *PRA* posterior retroperitoneoscopic adrenalectomy, *VAS* visual analog scale, *POD* postoperative day.

As shown in Table 3, the patients were divided into two groups according to tumor size: the small tumor group [< 3 cm, n = 74 (60.7%)] and large tumor group [≥ 3 cm, n = 48 (39.3%)]. The mean operation time was significantly longer for the large tumor group than for the small tumor group (160.3 ± 47.0 vs. 136.9 ± 47.9 min, *P* = 0.009). Pheochromocytoma in the large tumor group and primary hyperaldosteronism in the small tumor group were frequently diagnosed before surgery (*P* < 0.001). However, there were no statistically significant differences in age, sex, BMI, surgical approach, operative side, and postoperative VAS pain score.

**Table 3:** Baseline clinicopathological characteristics according to tumor size.

	Tumor size < 3 cm (n = 74)	Tumor size ≥ 3 cm (n = 48)	p-value
Age (years)	50.3 ± 11.8	50.2 ± 16.3	0.974
Gender			0.259
Male	27 (36.5%)	23 (47.9%)	
Female	47 (63.5%)	25 (52.1%)	
BMI (kg/m^2^)	24.9 ± 3.6	24.7 ± 4.9	0.856
Overweight	33 (44.6%)	19 (39.6%)	0.708
Operation time (min)	136.9 ± 47.9	160.3 ± 47.0	0.009
Surgical approach			0.701
LTA	68 (91.9%)	45 (93.8%)	
PRA	6 (8.1%)	3 (6.2%)	
Operation side			0.543
Right	20 (27.0%)	16 (33.3%)	
Left	54 (73.0%)	32 (66.7%)	
Surgical period			0.296
2009–2018	35 (47.3%)	28 (58.3%)	
2019–2022	39 (52.7%)	20 (41.7%)	
Preoperative diagnosis			< 0.001
Non-functioning lesions	45 (60.8%)	28 (58.3%)	
Primary hyperaldosteronism	16 (21.6%)	0 (0%)	
Pheochromocytoma	4 (5.4%)	16 (33.3%)	
Cushing’s syndrome	9 (12.2%)	4 (8.3%)	
Postoperative VAS			
POD 0	5.2 ± 1.6	4.9 ± 1.9	0.288
POD 1	3.5 ± 1.1	3.5 ± 1.1	0.846
POD 2	3.1 ± 0.9	3.1 ± 1.0	0.957
POD 3	2.8 ± 0.9	2.8 ± 0.9	0.751

Data were expressed as number (%) or mean ± standard deviation.

A statistically significant difference was defined as p < 0.05.

*BMI* body mass index, *LTA* lateral transperitoneal adrenalectomy, *PRA* posterior retroperitoneoscopic adrenalectomy, *VAS* visual analog scale, *POD* postoperative day.

The baseline clinicopathological characteristics according to BMI are presented in Table 4. The patients were divided into groups according to BMI: the normal group [≤ 25 kg/m^2^, n = 70 (57.4%)] and the overweight group [> 25 kg/m^2^, n = 52 (42.6%)]. There were no statistically significant differences in any of the baseline clinicopathological characteristics between the two groups.

**Table 4:** Baseline clinicopathological characteristics according to BMI.

	BMI ≤ 25 kg/m^2^ (n = 70)	BMI > 25 kg/m^2^ (n = 52)	**p-value**
Age (years)	51.2 ± 14.0	49.1 ± 13.2	0.412
Gender			0.355
Male	26 (37.1%)	24 (46.2%)	
Female	44 (62.9%)	28 (53.8%)	
Tumor size (cm)	2.9 ± 1.7	2.7 ± 1.1	0.520
Operation time (min)	143.9 ± 51.9	149.1 ± 44.5	0.559
Surgical approach			0.909
LTA	65 (92.9%)	48 (92.3%)	
PRA	5 (7.1%)	4 (7.7%)	
Operation side			0.163
Right	17 (24.3%)	19 (36.5%)	
Left	53 (75.7%)	33 (63.5%)	
Surgical period			0.029
2009–2018	30 (42.9%)	33 (63.5%)	
2019–2022	40 (57.1%)	19 (36.5%)	
Preoperative diagnosis			0.450
Non-functioning lesions	40 (57.1%)	33 (63.5%)	
Primary hyperaldosteronism	8 (11.4%)	8 (15.4%)	
Pheochromocytoma	12 (17.1%)	8 (15.4%)	
Cushing’s syndrome	10 (14.3%)	3 (5.8%)	
Postoperative VAS			
POD 0	5.0 ± 1.7	5.2 ± 1.8	0.618
POD 1	3.5 ± 1.1	3.5 ± 1.1	0.907
POD 2	3.2 ± 1.0	3.0 ± 0.9	0.239
POD 3	2.9 ± 0.9	2.7 ± 0.9	0.448

Data were expressed as number (%) or mean ± standard deviation.

A statistically significant difference was defined as p < 0.05.

*BMI* body mass index, *LTA* lateral transperitoneal adrenalectomy, *PRA* posterior retroperitoneoscopic adrenalectomy, *VAS* visual analog scale, *POD* postoperative day.

The results of the comparison of the baseline clinical characteristics according to the surgical period are summarized in Table 5. We divided the patients into two groups on the basis of the year 2019: the early group [to 2018, n = 63 (51.6%)] and late group [from 2019, n = 59 (48.4%)]. The late group was significantly older than the early group (54.2 ± 12.9 vs. 46.6 ± 13.4 years, *P* = 0.002). The mean operation time was significantly shorter in the late group than in the early group (128.9 ± 37.7 vs. 162.1 ± 52.6 min, *P* < 0.001). Since 2019, both LTA and PRA have been performed at our institution (*P* < 0.001). Compared with the early group, the late group underwent significantly more operations for functional diseases than for non-functioning lesions (*P* < 0.001). However, there were no statistically significant differences in sex, BMI, tumor size, operative side, and postoperative VAS pain score.

**Table 5:** Baseline clinicopathological characteristics according to surgical period.

	To 2018 (n = 63)	From 2019 (n = 59)	p-value
Age (years)	46.6 ± 13.4	54.2 ± 12.9	0.002
Gender			0.582
Male	24 (38.1%)	26 (44.1%)	
Female	39 (61.9%)	33 (55.9%)	
BMI (kg/m^2^)	25.3 ± 4.0	24.4 ± 4.4	0.230
Overweight	33 (55.9%)	19 (32.2%)	
Tumor size (cm)	2.9 ± 1.1	2.8 ± 1.8	0.694
Operation time (min)	162.1 ± 52.6	128.9 ± 37.7	< 0.001
Surgical approach			0.001
LTA	63 (100%)	50 (84.7%)	
PRA	0 (0%)	9 (15.3%)	
Operation side			0.871
Right	19 (30.2%)	17 (28.8%)	
Left	44 (69.8%)	42 (71.2%)	
Preoperative diagnosis			< 0.001
Non-functioning lesions	50 (79.4%)	23 (39.0%)	
Primary hyperaldosteronism	1 (1.6%)	15 (25.4%)	
Pheochromocytoma	12 (19.0%)	8 (13.6%)	
Cushing’s syndrome	0 (0%)	13 (22.0%)	
Postoperative VAS			
POD 0	5.2 ± 1.6	4.9 ± 1.8	0.356
POD 1	3.5 ± 1.2	3.5 ± 1.0	0.879
POD 2	3.1 ± 1.0	3.1 ± 0.9	0.677
POD 3	2.8 ± 0.8	2.9 ± 1.0	0.604

Data were expressed as number (%) or mean ± standard deviation.

A statistically significant difference was defined as p < 0.05.

*BMI* body mass index, *LTA* lateral transperitoneal adrenalectomy, *PRA* posterior retroperitoneoscopic adrenalectomy, *VAS* visual analog scale, *POD* postoperative day.

### Logistic regression analysis of risk factors associated with operation time

**Figure 1:**
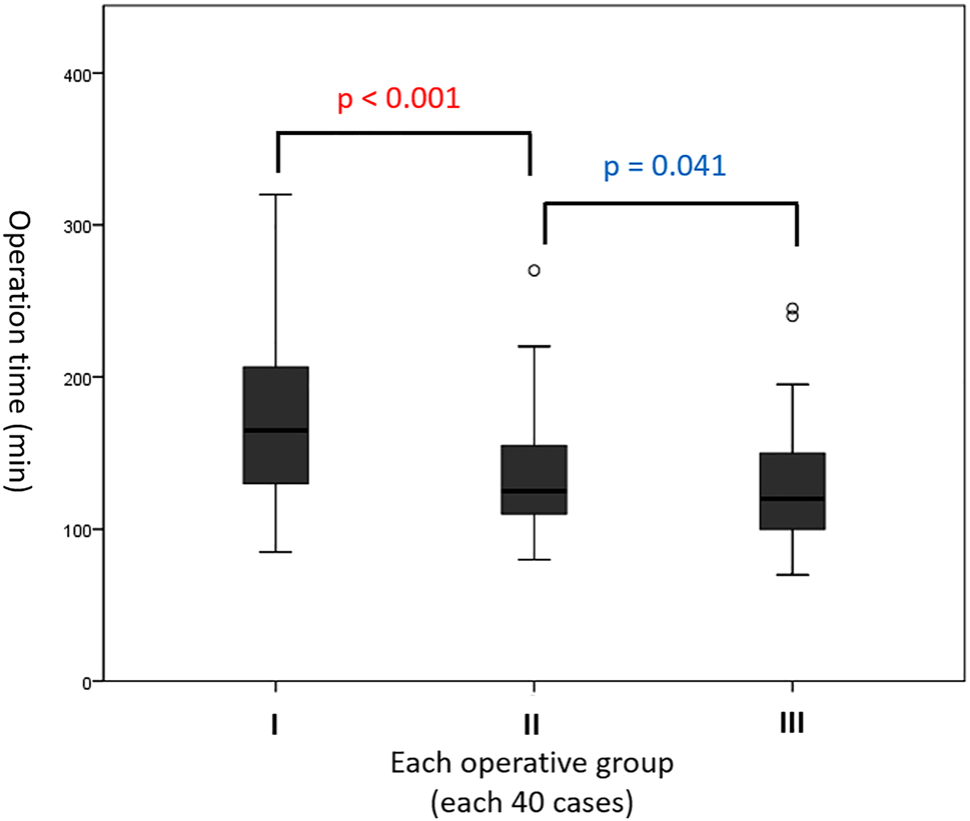
Mean operating time throughout three consecutive groups, which were 175.1 ± 54.1, 135.4 ± 37.8, and 128.7 ± 40.3 min, respectively, for groups I, II, and III. (I vs. II, *P* < 0.001, II vs. III, *P* = 0.441, I vs. III, *P* < 0.001).

We performed multivariate logistic regression analysis to validate the factors associated with operation time (Table 6). Univariate analysis identified tumor size, surgical period, primary hyperaldosteronism, and Cushing’s syndrome as significant risk factors for longer operation times. However, only pheochromocytoma was a significant independent risk factor for a longer operation time in the multivariate analysis (OR, 3.709; 95% CI, 1.127–12.205; *P* = 0.031).The operation time trend is shown in Fig. 1. The 122 adrenalectomy cases were divided into three groups, each comprising approximately 40 consecutive cases. The mean operation times for Groups I, II, and III were 175.1 ± 54.1, 135.4 ± 37.8, and 128.7 ± 40.3 min, respectively. There was a statistically significant decrease in the mean operation time between groups I and II (*P* < 0.001) and between groups I and III (*P* < 0.001).

**Table 6:** Logistic regression analysis of risk factors associated with long operation time > 150 min.

Variables	Simple generalized linear model			Multiple generalized linear model		
	OR	CI	p-value	OR	CI	p-value
Age	0.982	0.955–1.008	0.175			
Sex						
Female	Ref					
Male	2.000	0.954–4.191	0.066			
BMI(kg/m^2^)						
Overweight	1.168	0.562–2.426	0.677			
Tumor size (cm)	1.593	1.174–2.162	0.003			
< 3 cm	ref					
≥ 3 cm	3.039	1.425–6.481	0.004			
Surgical period						
2009–2018	Ref					
2019–2022	0.338	0.159–0.722	0.005			
Preoperative diagnosis						
Non-functioning lesions	Ref			Ref		
Primary hyperaldosteronism	8.372	1.033–67.864	0.047	0.680	0.149–3.117	0.620
Pheochromocytoma	2.769	0.252–30.383	0.405	3.709	1.127–12.205	0.031
Cushing’s syndrome	36.000	3.692–351.002	0.002	0.202	0.022–1.834	0.155

Data are expressed as Odds ratio (OR) and 95% confidence interval (CI).

A statistically significant difference was defined as p < 0.05.

*BMI* body mass index.

## Discussion

The adoption of RA for surgical management of adrenal gland diseases is a significant shift in the paradigm of endocrine surgery^1,3,4^. Our retrospective analysis of 122 consecutive cases adds valuable evidence to the increasing body of support for the safety and effectiveness of RA. The transition from open adrenalectomy to laparoscopic adrenalectomy marked a pivotal advancement in the field, and the evolution to RA now promises further refinement in technique and improvement in patient outcomes. Vatanserver et al. demonstrated that RA has lower complication rate and a shorter hospital stay compared to laparoscopic adrenalectomy^19^. Another study identified that despite the higher costs of RA, it appears to be cost-effective and economically sustainable in high-volume centers especially when performed for challenging cases^30^.

In our study of 122 consecutive cases from October 2009 to December 2022, we evaluated the safety and surgical outcomes of RA. Notably, all procedures were performed by a single surgeon with extensive experience in RA, ensuring a consistent and skilled approach. The baseline clinicopathological characteristics of our patient cohort reflect the diversity of adrenal gland diseases. The demographic profile of our study cohort reflects the prevalence of adrenal gland diseases in the population. The mean age of 50.3 years is in the typical age range for manifestation of these disorders, and the slightly higher representation of females (59%) is consistent with the existing literature suggesting a higher incidence of adrenal tumors in women^3,31^. Our study also encompassed a variety of adrenal disorders, with non-functioning lesions, primary hyperaldosteronism, pheochromocytoma, and Cushing’s syndrome accounting for 59.8%, 13.1%, 16.4%, and 10.7% of cases, respectively. This diversity underscores the versatility of RA in addressing a spectrum of adrenal pathologies.

Our analysis extensively explored various clinicopathological factors, providing insights into how they affect surgical outcomes. Sex differences were observed, with females having a shorter mean operation time than males. This observation raises intriguing questions about the anatomical variations and hormonal influences affecting the complexity of RA. Tumor size proved to be one of the key factors influencing the operation time, particularly as larger tumors required more intricate dissection, resulting in prolonged durations. The distribution of specific diagnoses among different tumor size groups highlights the importance of preoperative considerations in determining the most suitable surgical approach.

Our analysis also explored the effect of BMI on surgical outcomes. Notably, there were no statistically significant differences between the normal-weight and overweight groups, indicating that RA can be effectively performed across BMI categories. This result was consistent with that of a previous study by Agcaoglu^32^. This wide applicability is considered an advantage of RA, especially when considering the global rise in obesity rates and associated challenges in surgical interventions.

Although RA demonstrates overall safety and efficacy, our multivariate logistic regression analysis identified pheochromocytoma as a significant independent risk factor for longer operation times. This finding is consistent with the known challenges posed by vascularity and potential hemodynamic fluctuations associated with pheochromocytoma resection. Compared with other adrenal diseases, pheochromocytoma presents distinct challenges for surgeons that are primarily attributed to increased vascularity, firm texture, and its intricate relationship with the vasculature. Achieving surgical removal of these tumors with clear margins and without capsular disruption is crucial for preventing potential recurrences^33^. The challenges encountered during pheochromocytoma resection underscore the importance of meticulous preoperative planning and intraoperative management.

A temporal analysis revealed a progressive reduction in the mean operation time over the study period from October 2009 to December 2022. This positive trend can be attributed to various factors, including the surgeon’s accumulating experience with RA, advancements in robotic technology, and refined patient selection criteria. The observed decrease in operation time aligns with the broader trend in modern surgery, emphasizing the importance of minimizing procedure durations for improved patient safety and resource optimization.

Although our findings contribute valuable insights, several limitations should be acknowledged. The retrospective nature of the study introduces inherent biases, and the results may have been influenced by the surgeon’s experience and evolving practice patterns over the study duration. Prospective studies with larger sample sizes are warranted to further validate with our findings and explore additional advantages in the application of RA.

We highlight several limitations associated with RA. While surgeons experienced in laparoscopic adrenalectomy may swiftly adapt to RA, those lacking such experience may encounter obstacles in overcoming the learning curve. The posterior location of the adrenal gland within the abdomen presents anatomical complexities, particularly for surgeons unfamiliar with robotic instrumentation, thus representing a significant challenge. Additionally, RA in confined spaces, particularly when dealing with larger tumors, presents inherent challenges. Nevertheless, these challenges can be mitigated to some extent through the accumulation of experience and expertise. Furthermore, the issue of cost must be considered. The utilization of robotic systems in RA might have limitations in terms of cost-effectiveness, warranting careful evaluation of its financial implications. Despite the higher cost, RA is expected to have increased cost-effectiveness for larger and more challenging lesions^34^.

However, our study’s strengths lie in its single surgeon approach, which provides a homogeneous dataset reflective of the expertise of a seasoned practitioner. The inclusion of both LTA and PRA cases enabled a comprehensive comparison, offering insights into the evolving landscape of adrenal surgery.

## Conclusion

Our study contributes valuable insights into the safety and efficacy of RA. As technology continues to advance and surgical expertise improves further, RA is poised to have an increasingly prominent role in the management of adrenal gland diseases. Future research should focus on expanding the evidence base, refining surgical techniques, and assessing the long term outcomes of RA. Ultimately, integrating RA into routine clinical practice has the potential to revolutionize adrenal surgery, offering improved outcomes and enhanced patient care.

## Data Availability

The data that support the findings of this study are available on request from the corresponding author. The data are not publicly available due to privacy or ethical restrictions.

## References

[1] Makay O, Erol V, Ozdemir M (2019). Robotic adrenalectomy. Gland Surg..

[2] Ruhle BC, Ferguson Bryan A, Grogan RH (2019). Robot-assisted endocrine surgery: Indications and drawbacks. J. Laparoendosc. Adv. Surg. Tech..

[3] Economopoulos KP (2017). Laparoscopic versus robotic adrenalectomy: A comprehensive meta-analysis. Int. J. Surg..

[4] Grogan RH (2020). Current status of robotic adrenalectomy in the United States. Gland Surg..

[5] Sherlock M (2020). Adrenal incidentaloma. Endocr. Rev..

[6] Mihai R (2019). Open adrenalectomy. Gland Surg..

[7] Taffurelli G, Ricci C, Casadei R, Selva S, Minni F (2017). Open adrenalectomy in the era of laparoscopic surgery: A review. Update Surg..

[8] Gagner M (1992). Laparoscopic adrenalectomy in Cushing's syndrome and pheochromocytoma. N. Engl. J. Med..

[9] Gupta PK (2011). Outcomes after laparoscopic adrenalectomy. Surg. Endosc..

[10] Raffaelli M, De Crea C, Bellantone R (2019). Laparoscopic adrenalectomy. Gland Surg..

[11] Sommerey S (2015). Laparoscopic adrenalectomy—10-year experience at a teaching hospital. Langenbeck's Arch. Surg..

[12] Conzo G (2018). Single center experience with laparoscopic adrenalectomy on a large clinical series. BMC Surg..

[13] Barczyński M, Konturek A, Nowak W (2014). Randomized clinical trial of posterior retroperitoneoscopic adrenalectomy versus lateral transperitoneal laparoscopic adrenalectomy with a 5-year follow-up. Ann. Surg..

[14] Brandao LF (2014). Robotic versus laparoscopic adrenalectomy: A systematic review and meta-analysis. Eur. Urol..

[15] Samreen S (2019). Laparoscopic versus robotic adrenalectomy: A review of the national inpatient sample. J. Robot. Surg..

[16] Agrusa A (2017). Innovation in endocrine surgery: Robotic versus laparoscopic adrenalectomy. Meta-analysis and systematic literature review. Oncotarget.

[17] Yiannakopoulou E (2016). Robotic assisted adrenalectomy: Surgical techniques, feasibility, indications, oncological outcome and safety. Int. J. Surg..

[18] Perivoliotis K, Baloyiannis I, Sarakatsianou C, Tzovaras G (2020). Comparing the efficacy and safety of laparoscopic and robotic adrenalectomy: A meta-analysis and trial sequential analysis. Langenbeck's Arch. Surg..

[19] Vatansever S (2022). Robot-assisted versus conventional laparoscopic adrenalectomy: Results from the EUROCRINE surgical registry. Surgery.

[20] Sforza S (2021). Perioperative outcomes of robotic and laparoscopic adrenalectomy: A large international multicenter experience. Surg. Endosc..

[21] Gan, L. *et al.* Comparison of the effectiveness and safety of robotic-assisted and laparoscopic in adrenalectomy: A systematic review and meta-analysis. *Int. J. Surg.* 106853 (2022). 10.1016/j.ijsu.2022.106853 36075556

[22] James PT (2004). Obesity: The worldwide epidemic. Clin. Dermatol..

[23] Deitel M (2003). Overweight and obesity worldwide now estimated to involve 1.7 billion people. Obes. Surg..

[24] Fan JG, Kim SU, Wong VW (2017). New trends on obesity and NAFLD in Asia. J. Hepatol..

[25] Brunaud L (2008). Robotic-assisted adrenalectomy: What advantages compared to lateral transperitoneal laparoscopic adrenalectomy?. Am. J. Surg..

[26] You JY (2013). Comparison of robotic adrenalectomy with traditional laparoscopic adrenalectomy with a lateral transperitoneal approach: A single-surgeon experience. Int. J. Med. Robot..

[27] Nomine-Criqui C (2015). Robotic lateral transabdominal adrenalectomy. J. Surg. Oncol..

[28] Kim WW, Lee YM, Chung KW, Hong SJ, Sung TY (2019). Comparison of robotic posterior retroperitoneal adrenalectomy over laparoscopic posterior retroperitoneal adrenalectomy: A single tertiary center experience. Int. J. Endocrinol..

[29] Gokceimam M, Kahramangil B, Akbulut S, Erten O, Berber E (2021). Robotic posterior retroperitoneal adrenalectomy: Patient selection and long-term outcomes. Ann. Surg. Oncol..

[30] De Crea C (2022). Robot-assisted vs laparoscopic lateral transabdominal adrenalectomy: A propensity score matching analysis. Surg. Endosc..

[31] Pineda-Solís K, Medina-Franco H, Heslin MJ (2013). Robotic versus laparoscopic adrenalectomy: A comparative study in a high-volume center. Surg. Endosc..

[32] Agcaoglu O, Akbas M, Ozdemir M, Makay O (2019). The impact of body mass index on perioperative outcomes of robotic adrenalectomy: An update. Surg. Innov..

[33] Isiktas G (2022). Laparoscopic versus robotic adrenalectomy in pheochromocytoma patients. J. Surg. Oncol..

[34] De Crea C (2020). Robotic adrenalectomy: Evaluation of cost-effectiveness. Gland Surg..

